# 1-Benzyl-5-methyl-1*H*-1,2,3-triazole-4-carboxylic acid monohydrate

**DOI:** 10.1107/S160053680901678X

**Published:** 2009-05-14

**Authors:** Hong Zhao

**Affiliations:** a Ordered Matter Science Research Center, College of Chemistry and Chemical, Engineering, Southeast University, Nanjing 210096, People’s Republic of China

## Abstract

In the title compound, C_11_H_11_N_3_O_2_·H_2_O, the planes of the triazole and phenyl rings are almost perpendicular to each other [dihedral angle 89.5 (3)°]. The crystal packing is stabilized by strong inter­molecular O—H⋯O and O—H⋯N hydrogen bonds involving the water mol­ecule as both donor and acceptor.

## Related literature

For the synthesis of the title compound, see: El Khadem *et al.* (1968 ▶). For the biological activity of triazole compounds, see: Olesen *et al.* (2003 ▶); Tian *et al.* (2005 ▶). For related structures, see: Lin *et al.* (2008 ▶); Xiao *et al.* (2008 ▶).
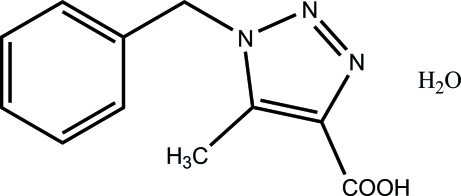


## Experimental

### 

#### Crystal data

C_11_H_11_N_3_O_2_·H_2_O
*M* *_r_* = 235.24
Triclinic, 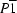

*a* = 6.5808 (13) Å
*b* = 7.4995 (15) Å
*c* = 12.337 (3) Å
α = 99.87 (4)°
β = 93.75 (3)°
γ = 91.80 (3)°
*V* = 598.0 (2) Å^3^
*Z* = 2
Mo *K*α radiation
μ = 0.10 mm^−1^
*T* = 292 K
0.35 × 0.30 × 0.25 mm

#### Data collection

Rigaku SCXmini diffractometer
Absorption correction: multi-scan (*CrystalClear*; Rigaku, 2005 ▶) *T*_min_ = 0.963, *T*_max_ = 0.976
6256 measured reflections
2730 independent reflections
1540 reflections with *I* > 2σ(*I*)
*R*_int_ = 0.042

#### Refinement

*R*[*F* ^2^ > 2σ(*F* ^2^)] = 0.064
*wR*(*F* ^2^) = 0.174
*S* = 1.05
2730 reflections
156 parameters
H-atom parameters constrained
Δρ_max_ = 0.19 e Å^−3^
Δρ_min_ = −0.15 e Å^−3^

### 

Data collection: *CrystalClear* (Rigaku, 2005 ▶); cell refinement: *CrystalClear*; data reduction: *CrystalClear*; program(s) used to solve structure: *SHELXS97* (Sheldrick, 2008 ▶); program(s) used to refine structure: *SHELXL97* (Sheldrick, 2008 ▶); molecular graphics: *SHELXTL* (Sheldrick, 2008 ▶); software used to prepare material for publication: *SHELXTL*.

## Supplementary Material

Crystal structure: contains datablocks I, New_Global_Publ_Block. DOI: 10.1107/S160053680901678X/rz2316sup1.cif

Structure factors: contains datablocks I. DOI: 10.1107/S160053680901678X/rz2316Isup2.hkl

Additional supplementary materials:  crystallographic information; 3D view; checkCIF report

## Figures and Tables

**Table 1:** Hydrogen-bond geometry (Å, °)

*D*—H⋯*A*	*D*—H	H⋯*A*	*D*⋯*A*	*D*—H⋯*A*
O1*W*—H1*A*⋯N1	0.92	1.96	2.870 (3)	172
O1*W*—H1*B*⋯O2^i^	0.86	1.88	2.734 (3)	171
O1—H1⋯O1*W*^ii^	0.82	1.75	2.563 (3)	168

Symmetry codes: (i) 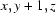; (ii) 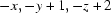.

**Fig. 1.**
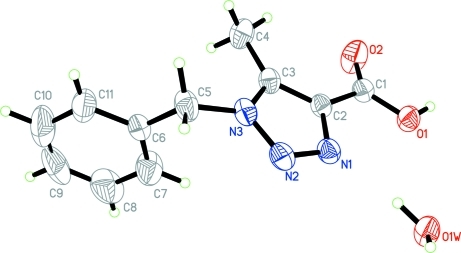
The molecular structure of the title compound, showing the atomic numbering scheme. Displacement ellipsoids are drawn at the 30% probability level.

**Fig. 2.**
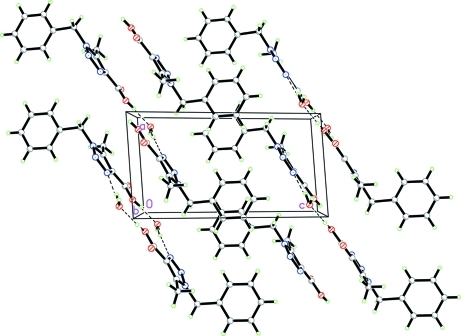
Packing diagram of the title compound viewed along the b axis. Intermolecular H bonds are shown as dashed lines.

## supplementary crystallographic information

### Comment

Triazole-related molecules have attracted considerable attention due to their biological activities (Olesen *et al.*, 2003; Tian *et al.*, 2005). Recently, we have reported the cerystal structure of a few triazole compounds (Lin *et al.* 2008; Xiao *et al.*2008). As an extension of our work on the structural characterization of the triazole-related compounds, we report herein the crystal structure of the title compound.

In the molecule of the title compound (Fig. 1), bond lengths and angles have normal values. The dihedral angle between the triazole and phenyl rings is 89.5 (3)°. The packing is stabilized by strong intermolecular O—H···O and O—H···N hydrogen bonds involving the triazole molecules and lattice water molecules (Fig.2; Table 1).

### Experimental

The title compound was prepared from azidomethylbenzene according to the reported method (El Khadem *et al.*, 1968). Colourless prismatic crystals suitable for X-ray analysis were obtained by slow evaporation of a 95% ethanol/water solution at room temperature.

### Refinement

The water and carboxylic H atoms were located from a difference Fourier map but not refined [*U*_iso_(H) = 1.5*U*_eq_(O)]. All other H atoms were fixed geometrically and treated as riding, with C—H = 0.93-0.97 Å and with *U*_iso_(H) = 1.2*U*_eq_(C) or 1.5 *U*_eq_(C) for methyl H atoms.

### Crystal data

**Table:**

C_11_H_11_N_3_O_2_·H_2_O	*Z* = 2
*M**_r_* = 235.24	*F*(000) = 248
Triclinic, *P*1	*D*_x_ = 1.306 Mg m^−^^3^
Hall symbol: -P 1	Mo *K*α radiation, λ = 0.71073 Å
*a* = 6.5808 (13) Å	Cell parameters from 1986 reflections
*b* = 7.4995 (15) Å	θ = 2.6–27.5°
*c* = 12.337 (3) Å	µ = 0.10 mm^−^^1^
α = 99.87 (4)°	*T* = 292 K
β = 93.75 (3)°	Prism, colourless
γ = 91.80 (3)°	0.35 × 0.30 × 0.25 mm
*V* = 598.0 (2) Å^3^	

### Data collection

**Table:**

Rigaku SCXmini diffractometer	2730 independent reflections
Radiation source: fine-focus sealed tube	1540 reflections with *I* > 2σ(*I*)
graphite	*R*_int_ = 0.042
Detector resolution: 13.6612 pixels mm^-1^	θ_max_ = 27.5°, θ_min_ = 3.1°
ω scans	*h* = −8→8
Absorption correction: multi-scan (*CrystalClear*; Rigaku, 2005)	*k* = −9→9
*T*_min_ = 0.963, *T*_max_ = 0.976	*l* = −15→15
6256 measured reflections	

### Refinement

**Table:**

Refinement on *F*^2^	Primary atom site location: structure-invariant direct methods
Least-squares matrix: full	Secondary atom site location: difference Fourier map
*R*[*F*^2^ > 2σ(*F*^2^)] = 0.064	Hydrogen site location: inferred from neighbouring sites
*wR*(*F*^2^) = 0.174	H-atom parameters constrained
*S* = 1.05	*w* = 1/[σ^2^(*F*_o_^2^) + (0.0599*P*)^2^ + 0.187*P*] where *P* = (*F*_o_^2^ + 2*F*_c_^2^)/3
2730 reflections	(Δ/σ)_max_ < 0.001
156 parameters	Δρ_max_ = 0.19 e Å^−^^3^
0 restraints	Δρ_min_ = −0.15 e Å^−^^3^

### Special details

**Table:**

Geometry. All e.s.d.'s (except the e.s.d. in the dihedral angle between two l.s. planes) are estimated using the full covariance matrix. The cell e.s.d.'s are taken into account individually in the estimation of e.s.d.'s in distances, angles and torsion angles; correlations between e.s.d.'s in cell parameters are only used when they are defined by crystal symmetry. An approximate (isotropic) treatment of cell e.s.d.'s is used for estimating e.s.d.'s involving l.s. planes.
Refinement. Refinement of *F*^2^ against ALL reflections. The weighted *R*-factor *wR* and goodness of fit *S* are based on *F*^2^, conventional *R*-factors *R* are based on *F*, with *F* set to zero for negative *F*^2^. The threshold expression of *F*^2^ > σ(*F*^2^) is used only for calculating *R*-factors(gt) *etc*. and is not relevant to the choice of reflections for refinement. *R*-factors based on *F*^2^ are statistically about twice as large as those based on *F*, and *R*- factors based on ALL data will be even larger.

### Fractional atomic coordinates and isotropic or equivalent isotropic displacement parameters (Å^2^)

**Table:**

	*x*	*y*	*z*	*U*_iso_*/*U*_eq_	
C1	0.2853 (4)	0.2569 (4)	0.9216 (2)	0.0526 (6)	
C2	0.4367 (4)	0.3462 (3)	0.86392 (19)	0.0484 (6)	
C3	0.6073 (4)	0.2769 (3)	0.8171 (2)	0.0531 (6)	
C4	0.6958 (5)	0.0956 (4)	0.8063 (3)	0.0767 (9)	
H4A	0.6878	0.0398	0.7300	0.115*	
H4B	0.6210	0.0212	0.8473	0.115*	
H4C	0.8359	0.1084	0.8346	0.115*	
C5	0.8767 (4)	0.4291 (5)	0.7208 (2)	0.0715 (8)	
H5A	0.9851	0.3738	0.7586	0.086*	
H5B	0.9167	0.5557	0.7242	0.086*	
C6	0.8532 (4)	0.3374 (4)	0.6016 (2)	0.0609 (7)	
C7	0.6735 (6)	0.3212 (6)	0.5403 (3)	0.0988 (12)	
H7	0.5557	0.3604	0.5729	0.119*	
C8	0.6638 (8)	0.2462 (7)	0.4289 (3)	0.1207 (15)	
H8	0.5399	0.2379	0.3872	0.145*	
C9	0.8314 (11)	0.1859 (6)	0.3814 (3)	0.1187 (17)	
H9	0.8239	0.1327	0.3073	0.142*	
C10	1.0096 (9)	0.2029 (6)	0.4414 (5)	0.1221 (16)	
H10	1.1271	0.1639	0.4083	0.146*	
C11	1.0215 (5)	0.2769 (5)	0.5510 (3)	0.0872 (10)	
H11	1.1467	0.2860	0.5916	0.105*	
N1	0.4242 (3)	0.5221 (3)	0.85113 (17)	0.0557 (6)	
N2	0.5786 (4)	0.5666 (3)	0.79918 (18)	0.0624 (6)	
N3	0.6905 (3)	0.4179 (3)	0.77838 (16)	0.0567 (6)	
O1	0.1510 (3)	0.3660 (2)	0.96493 (16)	0.0649 (6)	
H1	0.0776	0.3125	1.0012	0.097*	
O2	0.2875 (4)	0.0973 (3)	0.9257 (2)	0.0921 (8)	
O1W	0.1065 (3)	0.7618 (2)	0.91803 (16)	0.0709 (6)	
H1A	0.2137	0.6876	0.9038	0.106*	
H1B	0.1686	0.8670	0.9279	0.106*	

### Atomic displacement parameters (Å^2^)

**Table:**

	*U*^11^	*U*^22^	*U*^33^	*U*^12^	*U*^13^	*U*^23^
C1	0.0594 (16)	0.0472 (15)	0.0502 (15)	−0.0021 (12)	0.0062 (12)	0.0056 (11)
C2	0.0559 (15)	0.0463 (13)	0.0418 (13)	−0.0018 (11)	0.0051 (11)	0.0043 (10)
C3	0.0562 (15)	0.0564 (16)	0.0425 (14)	−0.0037 (12)	0.0033 (11)	−0.0016 (11)
C4	0.079 (2)	0.0675 (19)	0.079 (2)	0.0093 (16)	0.0153 (16)	−0.0065 (16)
C5	0.0516 (16)	0.108 (2)	0.0536 (17)	−0.0118 (15)	0.0079 (13)	0.0128 (16)
C6	0.0641 (18)	0.0689 (18)	0.0531 (16)	0.0011 (14)	0.0143 (14)	0.0162 (13)
C7	0.080 (2)	0.148 (4)	0.059 (2)	0.008 (2)	−0.0022 (18)	−0.007 (2)
C8	0.142 (4)	0.149 (4)	0.062 (2)	−0.002 (3)	−0.012 (3)	0.003 (2)
C9	0.202 (6)	0.096 (3)	0.058 (2)	−0.009 (3)	0.048 (3)	0.003 (2)
C10	0.147 (4)	0.114 (3)	0.108 (4)	0.016 (3)	0.072 (3)	0.001 (3)
C11	0.083 (2)	0.092 (2)	0.090 (3)	0.0129 (19)	0.0349 (19)	0.013 (2)
N1	0.0634 (14)	0.0531 (13)	0.0527 (13)	−0.0007 (11)	0.0119 (10)	0.0125 (10)
N2	0.0677 (15)	0.0653 (15)	0.0570 (14)	−0.0054 (12)	0.0119 (11)	0.0172 (11)
N3	0.0560 (13)	0.0677 (15)	0.0446 (12)	−0.0065 (11)	0.0065 (10)	0.0052 (10)
O1	0.0683 (13)	0.0601 (11)	0.0732 (13)	0.0066 (10)	0.0273 (10)	0.0220 (9)
O2	0.1035 (17)	0.0458 (12)	0.134 (2)	−0.0006 (11)	0.0516 (15)	0.0183 (12)
O1W	0.0752 (13)	0.0540 (11)	0.0874 (14)	−0.0015 (10)	0.0318 (11)	0.0138 (10)

### Geometric parameters (Å, °)

**Table:**

C1—O2	1.208 (3)	C6—C11	1.359 (4)
C1—O1	1.302 (3)	C7—C8	1.389 (5)
C1—C2	1.470 (3)	C7—H7	0.9300
C2—N1	1.360 (3)	C8—C9	1.338 (6)
C2—C3	1.372 (3)	C8—H8	0.9300
C3—N3	1.347 (3)	C9—C10	1.336 (6)
C3—C4	1.485 (4)	C9—H9	0.9300
C4—H4A	0.9600	C10—C11	1.366 (6)
C4—H4B	0.9600	C10—H10	0.9300
C4—H4C	0.9600	C11—H11	0.9300
C5—N3	1.463 (3)	N1—N2	1.301 (3)
C5—C6	1.509 (4)	N2—N3	1.353 (3)
C5—H5A	0.9700	O1—H1	0.8200
C5—H5B	0.9700	O1W—H1A	0.9192
C6—C7	1.352 (4)	O1W—H1B	0.8630
			
O2—C1—O1	124.7 (2)	C11—C6—C5	119.0 (3)
O2—C1—C2	122.0 (2)	C6—C7—C8	120.4 (4)
O1—C1—C2	113.3 (2)	C6—C7—H7	119.8
N1—C2—C3	109.0 (2)	C8—C7—H7	119.8
N1—C2—C1	122.0 (2)	C9—C8—C7	120.4 (4)
C3—C2—C1	129.0 (2)	C9—C8—H8	119.8
N3—C3—C2	103.5 (2)	C7—C8—H8	119.8
N3—C3—C4	123.9 (2)	C10—C9—C8	119.3 (4)
C2—C3—C4	132.6 (2)	C10—C9—H9	120.3
C3—C4—H4A	109.5	C8—C9—H9	120.3
C3—C4—H4B	109.5	C9—C10—C11	120.8 (4)
H4A—C4—H4B	109.5	C9—C10—H10	119.6
C3—C4—H4C	109.5	C11—C10—H10	119.6
H4A—C4—H4C	109.5	C6—C11—C10	121.0 (4)
H4B—C4—H4C	109.5	C6—C11—H11	119.5
N3—C5—C6	113.3 (2)	C10—C11—H11	119.5
N3—C5—H5A	108.9	N2—N1—C2	109.1 (2)
C6—C5—H5A	108.9	N1—N2—N3	106.8 (2)
N3—C5—H5B	108.9	C3—N3—N2	111.6 (2)
C6—C5—H5B	108.9	C3—N3—C5	129.6 (3)
H5A—C5—H5B	107.7	N2—N3—C5	118.8 (2)
C7—C6—C11	117.9 (3)	C1—O1—H1	109.5
C7—C6—C5	123.0 (3)	H1A—O1W—H1B	100.8

### Hydrogen-bond geometry (Å, °)

**Table:**

*D*—H···*A*	*D*—H	H···*A*	*D*···*A*	*D*—H···*A*
O1W—H1A···N1	0.92	1.96	2.870 (3)	172
O1W—H1B···O2^i^	0.86	1.88	2.734 (3)	171
O1—H1···O1W^ii^	0.82	1.75	2.563 (3)	168

Symmetry codes: (i) *x*, *y*+1, *z*; (ii) −*x*, −*y*+1, −*z*+2.
